# Hepatitis E virus ORF3 protein hijacking thioredoxin domain-containing protein 5 (TXNDC5) for its stability to promote viral particle release

**DOI:** 10.1128/jvi.01649-23

**Published:** 2024-03-29

**Authors:** Yamin Sheng, Yingying Deng, Xiaoxuan Li, Pinpin Ji, Xuwen Sun, Baoyuan Liu, Jiahong Zhu, Jiakai Zhao, Yuchen Nan, En-Min Zhou, Julian A. Hiscox, James P. Stewart, Yani Sun, Qin Zhao

**Affiliations:** 1 Department of Preventive Veterinary Medicine, College of Veterinary Medicine, Northwest A&F University, Yangling, Shaanxi, China; 2 Department of Infection Biology and Microbiomes, Institute of Infection, Veterinary and Ecological Sciences, University of Liverpool, Liverpool, United Kingdom; University of Southern California, Los Angeles, California, USA

**Keywords:** hepatitis E virus, TXNDC5, virus–host interaction, ORF3 protein, virus release, protein stability and modification

## Abstract

Hepatitis E virus (HEV) is the most common cause of acute viral hepatitis worldwide, responsible for approximately 20 million infections annually. Among the three open reading frames (ORFs) of the HEV genome, the ORF3 protein is involved in virus release. However, the host proteins involved in HEV release need to be clarified. In this study, a host protein, thioredoxin domain-containing protein 5 (TXNDC5), interacted with the non-palmitoylated ORF3 protein by co-immunoprecipitation analysis. We determined that the overexpression or knockdown of TXNDC5 positively regulated HEV release from the host cells. The ^17^FCL^19^ mutation of the ORF3 protein lost the ability to interact with TXNDC5. The releasing amounts of HEV with the ORF3 mutation (FCL17-19SSP) were decreased compared with wild-type HEV. The overexpression of TXNDC5 can stabilize and increase ORF3 protein amounts, but not the TXNDC5 mutant with amino acids 1–88 deletion. Meanwhile, we determined that the function of TXNDC5 on the stabilization of ORF3 protein is independent of the Trx-like domains. Knockdown of TXNDC5 could lead to the degradation of ORF3 protein by the endoplasmic reticulum (ER)-associated protein degradation-proteasome system. However, the ORF3 protein cannot be degraded in the knockout-TXNDC5 stable cells, suggesting that it may hijack other proteins for its stabilization. Subsequently, we found that the other members of protein disulfide isomerase (PDI), including PDIA1, PDIA3, PDIA4, and PDIA6, can increase ORF3 protein amounts, and PDIA3 and PDIA6 interact with ORF3 protein. Collectively, our study suggested that HEV ORF3 protein can utilize TXNDC5 for its stability in ER to facilitate viral release.

**IMPORTANCE:**

Hepatitis E virus (HEV) infection is the leading cause of acute viral hepatitis worldwide. After the synthesis and modification in the cells, the mature ORF3 protein is essential for HEV release. However, the host protein involved in this process has yet to be determined. Here, we reported a novel host protein, thioredoxin domain-containing protein 5 (TXNDC5), as a chaperone, contributing to HEV release by facilitating ORF3 protein stability in the endoplasmic reticulum through interacting with non-palmitoylated ORF3 protein. However, we also found that in the knockout-TXNDC5 stable cell lines, the HEV ORF3 protein may hijack other proteins for its stabilization. For the first time, our study demonstrated the involvement of TXNDC5 in viral particle release. These findings provide some new insights into the process of the HEV life cycle, the interaction between HEV and host factors, and a new direction for antiviral design.

## INTRODUCTION

Hepatitis E virus (HEV) infection is the leading cause of acute viral hepatitis (1), responsible for approximately 20 million infections annually with an estimated 3.3 million symptomatic cases, and results in about 44,000 deaths (2). HEV is mainly transmitted through the fecal–oral route and usually becomes a self-limiting disease in healthy people, chronic infection in immunocompromised patients, and stillbirth in pregnant women (3). HEV, belonging to the *Hepeviridae* family, is a positive-sense single-stranded RNA and forms quasi-envelope particles in blood or tissue culture and non-envelope particles in feces (4–6). For the eight main genotypes of the *Paslahepevirus balayani* genera, genotypes 1–4 and 7 are considered human pathogenic strains (4). Genotype 1 and 2 HEVs (HEV-1 and HEV-2) only infect humans, genotype 3 and 4 HEVs (HEV-3 and HEV-4) cause zoonotic diseases, and genotype 7 (HEV-7) was hitherto detected in only one patient regularly taking camel milk and meat (7–9). The full length of HEV is approximately 7.2 kb and consists of three open reading frames (ORFs), named ORF1, ORF2, and ORF3, while the fourth ORF has been only identified in HEV-1 and overlaps with ORF1 (10). Specifically, ORF1 is translated directly from HEV genomic RNA and encodes non-structural proteins. ORF2 and ORF3 are translated from 2.2-kb subgenomic RNA generated by negative sense HEV RNA (11). The ORF3 partially overlaps with ORF2 and encodes a multifunctional, phosphorylated, and palmitoylation protein consisting of 113 or 114 amino acids (aa) (12, 13).

The egress of HEV requires the exosomal pathway, with secretory exosomes derived from multivesicular bodies (14). This process is assisted by the host factor of tumor suppressor gene 101 (TSG101), which is a member of the endosomal sorting complexes required for the transport pathway and interacts with the PSAP motif of ORF3 protein (15). The ORF3 protein forms the structure of multimeric complexes associated with endoplasmic reticulum (ER)-derived membranes and shares multiple features with class I viroporins (16). After assembling, ORF3 protein acts as an ion channel and is required to release infectious virions (16). Moreover, the N-terminus of the ORF3 protein is highly conserved and rich in cysteine, which determines the palmitoylation of the ORF3 protein (13). This modification is critical for membrane localization of ORF3 protein and is involved in HEV particle release (13). Therefore, it was well documented that HEV ORF3 protein plays a critical role in viral particle release (17, 18).

Additionally, several host factors have been characterized to interact with HEV ORF3 protein. For example, TSG101 can interact with ORF3 protein PSAP motifs and play a critical role in HEV secretion (15). The ORF3 protein can enhance the secretion of α1-microglobulin from the hepatocyte through its PSAP motif. The α1-microglobulin, TSG101, and ORF3 can be co-precipitated as a ternary complex (19, 20). Hemopexin was found to interact with the N-terminal hydrophobic domain II of ORF3 protein (21). ORF3 protein can lead to the elevation of acetylated alpha-tubulin by interacting with the microtubules, which promote microtubule stability (22). It was also found that the proline-rich region of ORF3 protein is the interacting site for src homology 3 domain-containing proteins, and they play an essential role in HEV release (23). These host proteins perform different functions when HEVs infect host cells. Nevertheless, the host proteins being involved in the stability of HEV ORF3 protein in the cells still need to be clarified.

The viral proteins usually hijack host ER factors to form the disulfide bonds (-s-s- bonds) between cysteine residues for correct folding, which helps viral proteins evade the degradation by the host proteasome system (24). As we know, the protein disulfide isomerase (PDI) family is involved in the catalysis of this process (25, 26). The PDI family consists of more than 20 members (26). Most contain one or more thioredoxin (Trx)-like domains to catalyze disulfide bond oxidation, reduction, and isomerization (27). Much evidence suggests that PDIs affect viral infection (28–31). For example, PDIA3 inhibits viral entry by the inhibition of *Zaire* ebolavirus structural glycoprotein expression (30), and PDIA4 is involved in human astrovirus infection by interacting with capsid spikes (31). The thioredoxin domain-containing protein 5 (TXNDC5) is also a member of the PDI family with three Trx-like domains (32). TXNDC5 contributes to correct cellular protein folding and enhances its stability, while the misfolded proteins are degraded through the proteasome system (33). Additionally, TXNDC5 mediates redox reaction by interacting with NADPH (34). However, whether the viral proteins hijack the TXNDC5 for its stability has yet to be reported.

In this study, the TXNDC5 was determined to interact with HEV ORF3 protein and maintain the stability of ORF3 protein. Then, it facilitates HEV release from the cells. However, the levels of HEV ORF3 proteins cannot be reduced in the TXNDC5^−/−^ stable cell lines (10 generations), suggesting that the ORF3 protein may hijack other proteins for its stabilization in the cells. Subsequently, we found that the PDIA1, PDIA3, PDIA4, and PDIA6 from the PDI family can also increase the ORF3 protein amounts, while PDIA3 and PDIA6 interact with HEV ORF3 protein. These findings provide new insights into the process of HEV infection and a novel therapy to control HEV infection.

## RESULTS

### Screening of host proteins being involved in HEV infection

**Fig 1:**
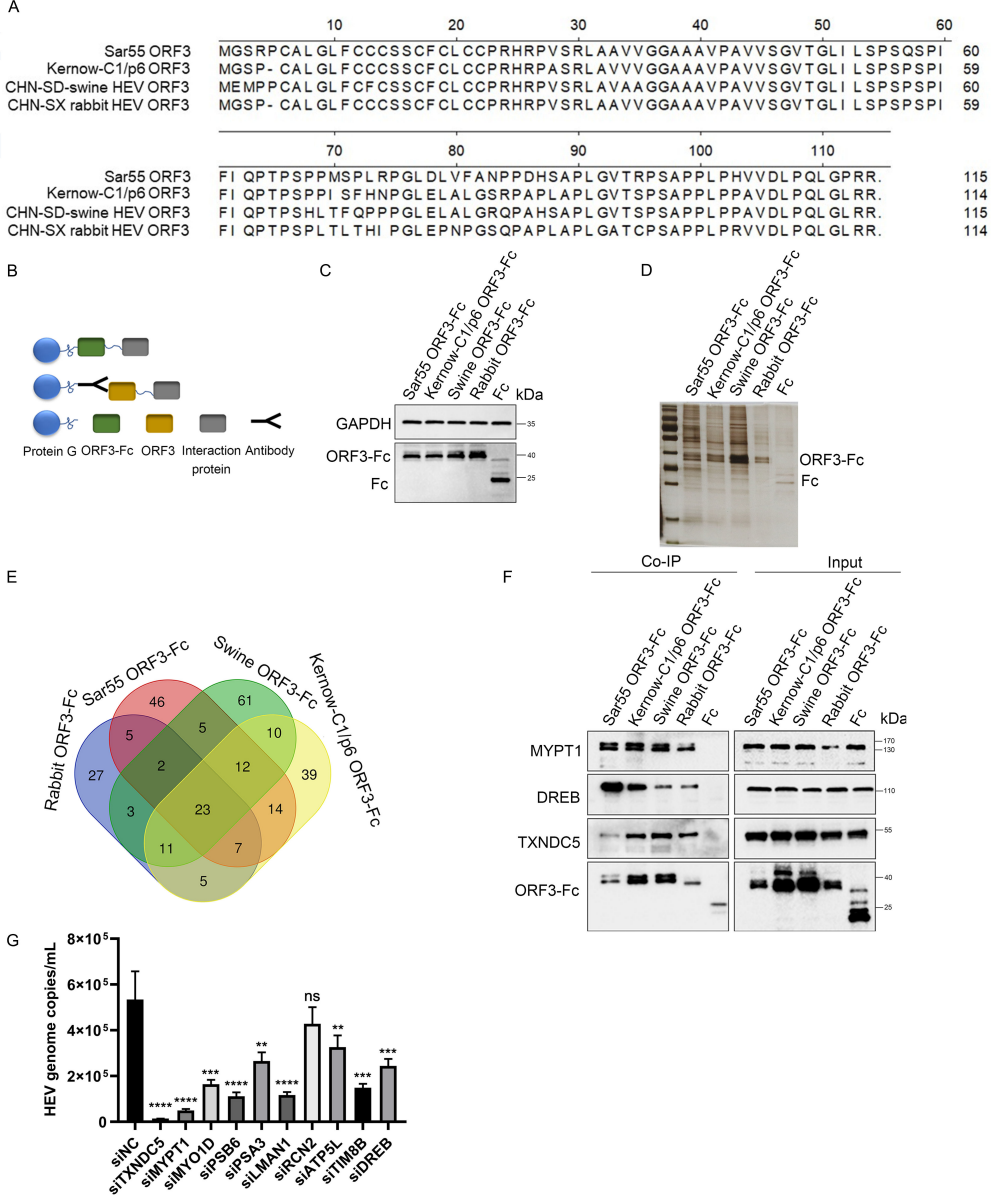
Screening and identification of cellular proteins pulled down by four HEV ORF3 proteins from three genotypes. (**A**) Sequence analysis of four HEV ORF3 proteins. Amino acid sequences of ORF3 (GenBank accession numbers AF444002, JQ679013, KF176351.1, and KX227751) were aligned using MegAlign software. (**B**) Schematic diagram of co-immunoprecipitation experiment. (**C**) Western blotting analysis of ORF3 protein fused with Fc and His tags or only Fc and His tags expressed in HEK293T cells. (**D**) After the HEK293T cells were separately transfected with four recombinant plasmids containing the genes encoding ORF3-Fc or Fc for 48 h, the cell lysates were immunoprecipitated with protein G. The silver staining showed that compared with the Fc tag, some cellular proteins were pulled down by the four ORF3 proteins. (**E**) The Venn diagram was analyzed by the website (https://bioinformatics.psb.ugent.be/webtools/Venn/). (**F**) Four HEV ORF3 proteins interacted with endogenous MYPT1, DREB, and TXNDC5. The immunoprecipitation mixture was analyzed by Western blotting with anti-MYPT1, anti-DREB, or anti-TXNDC5 antibodies. (**G**) Function analysis of the selected 10 host proteins in HEV release. The HepG2/C3A-p6 cells were transfected with indicated siRNAs for 48 h, and the supernatants were collected and analyzed by quantitative real-time PCR (RT-qPCR). Data represent means ± SDs of five biological repeats. ns, not significant; ^**^P < 0.01; ^***^P < 0.001; ^****^P < 0.0001.

Different genotypes of HEV ORF3 proteins consist of 113 or 114 aa with the predicted molecular weight (MW) of 13 kDa. The amino acid alignments of four HEV ORF3 proteins from three genotypes, including human HEV-1 (Sar55), human HEV-3 (Kernow-C1/p6), rabbit HEV-3 (CHN-SX-rHEV), and swine HEV-4 (CHN-SD-sHEV), showed that they shared 77.4%–86.8% identities (Fig. 1A). The four proteins were used as the baits for the co-immunoprecipitation (Co-IP) assay. Because the Fc fragment of IgG can directly bind to protein G, the above four ORF3 proteins fused with Fc, and His tags were designed (Fig. 1B) and successfully expressed in HEK293T cells with approximately expected sizes of 37 kDa (Fig. 1C). Then, using the four recombinant proteins and only Fc tag as the baits for the Co-IP assay, the results of silver staining showed that compared with the Fc tag, some cellular factors were pulled down by the four ORF3 proteins (Fig. 1D). Additionally, the immunoprecipitated complex proteins were separately analyzed with liquid chromatography coupled to tandem mass spectrometry (LC-MS/MS). Subsequently, the host proteins associated with ORF3 proteins were screened by comparing them with the only Fc tag. The results showed that 121 host proteins were pulled down by human HEV-3 ORF3 protein (g3-ORF3), 114 proteins by human HEV-1 ORF3 protein (g1-ORF3), 127 proteins by swine HEV-4 ORF3 protein (g4-ORF3), and 83 proteins by rabbit HEV-3 ORF3 protein (g3-rabbit-ORF3). Among these proteins, 23 host proteins were associated with all four ORF3 proteins (three genotypes) (Fig. 1E; Table S1). To confirm the results of LC-MS/MS, three proteins (MTPT1, DREB, and TXNDC5) were randomly selected further to confirm the interactions with HEV ORF3 proteins by Co-IP (Fig. 1F).

The previous study reported that ORF3 protein is associated with HEV release (16). Therefore, out of 23 host proteins, 10 (TXNDC5, MYPT1, MYO1D, PSB6, PSA3, LMAN1, RCN2, ATP5L, TIM8B, and DREB) were randomly selected to analyze whether they were involved in HEV release. Initially, siRNA duplexes were separately synthesized and used to knock down the above 10 proteins in HepG2/C3A-p6 cells. The HEV RNA in the supernatants was detected, and absolute quantitative RT-PCR results showed that the TXNDC5 was the most effective protein in inhibiting viral particle release (Fig. 1G). Therefore, TXNDC5 was selected for further study.

### TXNDC5 interacts with HEV ORF3 proteins

**Fig 2:**
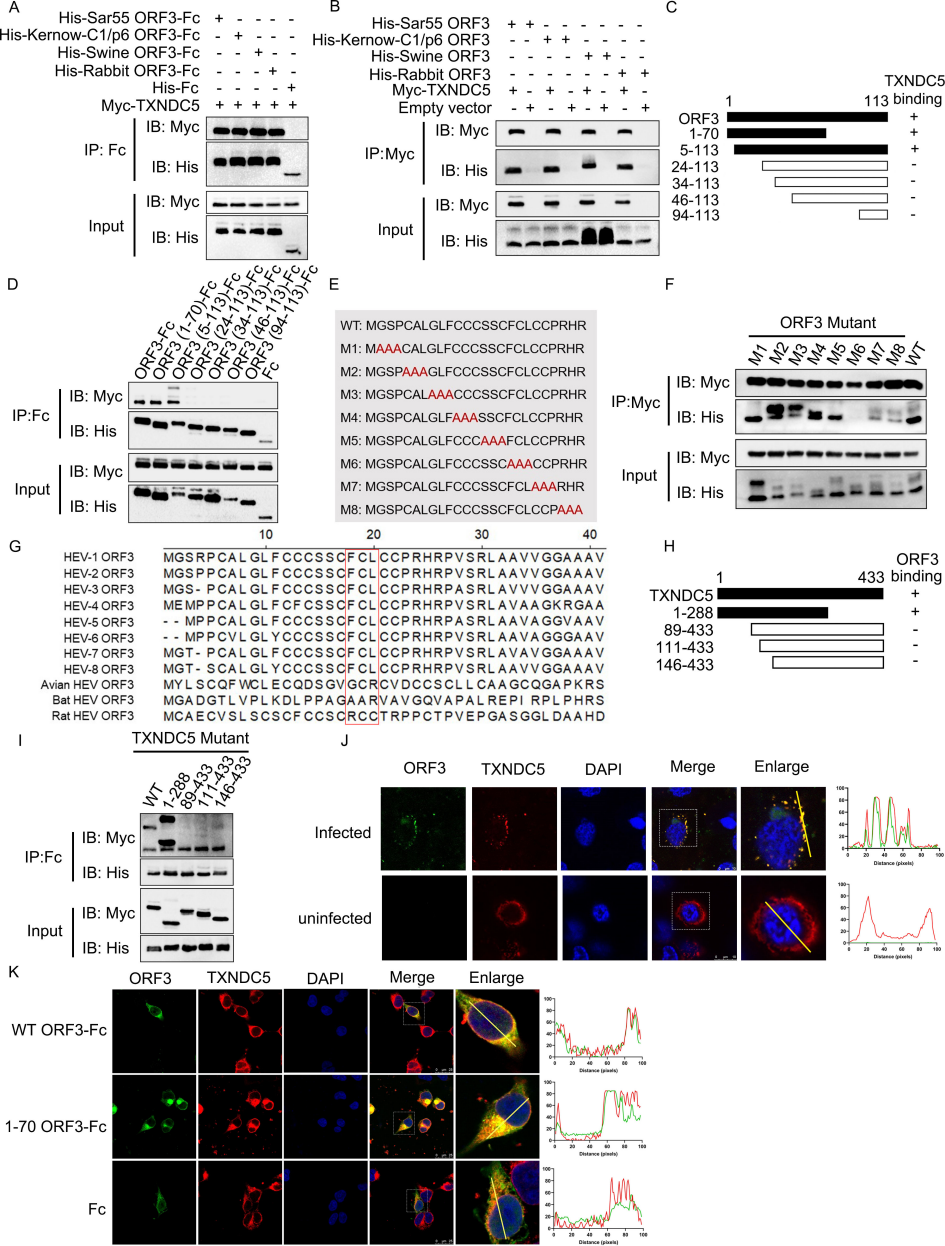
Mapping domain and motifs involved in the interaction between TXNDC5 and HEV ORF3 protein. (**A**) TXNDC5 can be pulled down by four HEV ORF3 proteins. HEK293T cells were transiently co-transfected with Myc-TXNDC5 and four His-ORF3-Fc or Fc plasmids. Then, the transfected cell lysates were subjected to immunoprecipitation with protein G, and the complex was analyzed by Western blotting with anti-His or anti-Myc antibodies. (**B**) Four HEV ORF3 proteins can be pulled down by the TXNDC5. HEK293T cells were co-transfected with only four His tag-ORF3 and Myc-TXNDC5 plasmids, the cell lysates were immunoprecipitated with an anti-Myc monoclonal antibody (mAb), and then, the complex was analyzed by Western blotting with the indicated antibodies. (**C**) Schematic diagram of the different truncated g3-ORF3 proteins, including aa 1–70, 5–113, 24–113, 34–113, 46–113, and 94–113. (**D**) The N-terminal of ORF3 (5–24) was the key domain of ORF3 protein interacting with TXNDC5. HEK293T was co-transfected with the plasmids containing the genes encoding Myc-TXNDC5 and different truncated g3-ORF3-Fc proteins. The cell lysates were then subjected to Co-IP, and the complex was analyzed by Western blotting. (**E**) Schematic diagram of the mutated g3-ORF3 for changing triple aa to alanine. (**F**) The ^17^FCL^19^ of ORF3 mutation does not interact with TXNDC5. Western blotting analysis of the Co-IP complex forms the HEK293T cells co-expressing Myc-TXNDC5 and different g3-ORF3 mutants. (**G**) Sequence analysis of ^17^FCL^19^ regions of four HEV ORF3 proteins. Amino acid sequences of ORF3 proteins (GenBank accession numbers M73218, KX578717, JQ679013, AJ272108, AB573435, AB602441, KJ496143, KX387865, GU95AA30, JQ001749, and GU345042) were aligned. (**H**) Schematic diagram of the different truncated TXNDC5 proteins. (**I**) The N-terminal of TXNDC5 (1–88) is the key domain for the interaction between TXNDC5 and ORF3 proteins. Western blotting analysis of the Co-IP complex from the HEK293T cells expressing g3-ORF3-Fc and different truncated TXNDC5 proteins. (**J**) TXNDC5 co-localized with ORF3 protein in Kernow/C1-p6-infected cells. After infection with Kernow/C1-p6, the cells were fixed and stained with anti-ORF3 mAb and TXNDC5 polyclonal antibody (pAb) and analyzed by confocal microscopy. Scale bars indicated 10 µm. (**K**) TXNDC5 co-localized with complete and the N-terminal of ORF3 protein. After transfection of His-ORF3_1-113_-Fc, His-ORF3_1-70_-Fc, and His-Fc plasmids, respectively, the cells were fixed and stained with anti-His mAb and TXNDC5 pAb. The location of ORF3 protein and TXNDC5 was analyzed by confocal microscopy. Scale bars indicated 25 µm. The profiles of fluorescence intensity along the yellow line in the enlarged images are shown in the right panels and analyzed using ImageJ software.

To further confirm the interaction between TXNDC5 and ORF3 protein, a Co-IP assay was conducted by transient co-expression of Myc-tagged TXNDC5 and His-tagged ORF3-Fc fusion proteins. The results showed that TXNDC5 could be pulled down by the four HEV ORF3 proteins rather than the Fc tag (Fig. 2A). In addition, the reverse Co-IP assay also revealed that four ORF3 proteins can be pulled down by the TXNDC5 (Fig. 2B).

To map the individual regions of the ORF3 protein responsible for the interaction with TXNDC5, the different truncated HEV-3 ORF3 proteins were designed and performed. Firstly, six truncated ORF3 fragments with Fc and His tags were designed (Fig. 2C). TXNDC5 and complete or truncated ORF3 proteins with Fc tag were transiently co-expressed in the HEK293T cells. The results of the Co-IP assay showed that the N-terminal region of ORF3 protein, including aa residues 5–24, was the domain for its interaction with TXNDC5 (Fig. 2D). Next, the mutant ORF3 proteins with the triple aa changed to alanine in the N-terminal region were designed (Fig. 2E) and used to determine the aa being responsible for the interaction. The Co-IP results showed that M6 mutant (comprising aa residues 17–19) lost the ability to interact with TXNDC5 (Fig. 2F). Additionally, the alignments of the ^17^FCL^19^ motifs of HEV ORF3 protein showed that the three motifs are conserved among eight genotypes of *Paslahepevirus balayani*, but not in avian, rat, and bat HEVs (Fig. 2G).

To further confirm the functional region in TXNDC5 for its interaction with ORF3 protein, a series of truncated and Myc-tagged TXNDC5 were designed (Fig. 2H). The results showed that deleting 1–88 aa in TXNDC5 abolished its interaction with ORF3 protein (Fig. 2I).

Next, to analyze the co-localization of TXNDC5 and HEV ORF3 proteins in the cells, confocal microscopy experiments were performed, and the results revealed that the TXNDC5 (red) was co-localized with ORF3 protein (green) in the Kernow-C1/p6-infected cells (Fig. 2J). Compared with the distributions of TXNDC5 in the normal cells, the TXNDC5 clustered into dots by interacting with ORF3 proteins in the HEV-infected cells (Fig. 2J). Additionally, the co-localization of the TXNDC5 with the full-length or N-terminal region of ORF3 protein in the cells was also analyzed, and the results showed that TXNDC5 (red) was co-localized with ORF3_1-113_-Fc and ORF3_1-70_-Fc proteins (green), but not with Fc tag (Fig. 2K).

### TXNDC5 is involved in the HEV release

**Fig 3:**
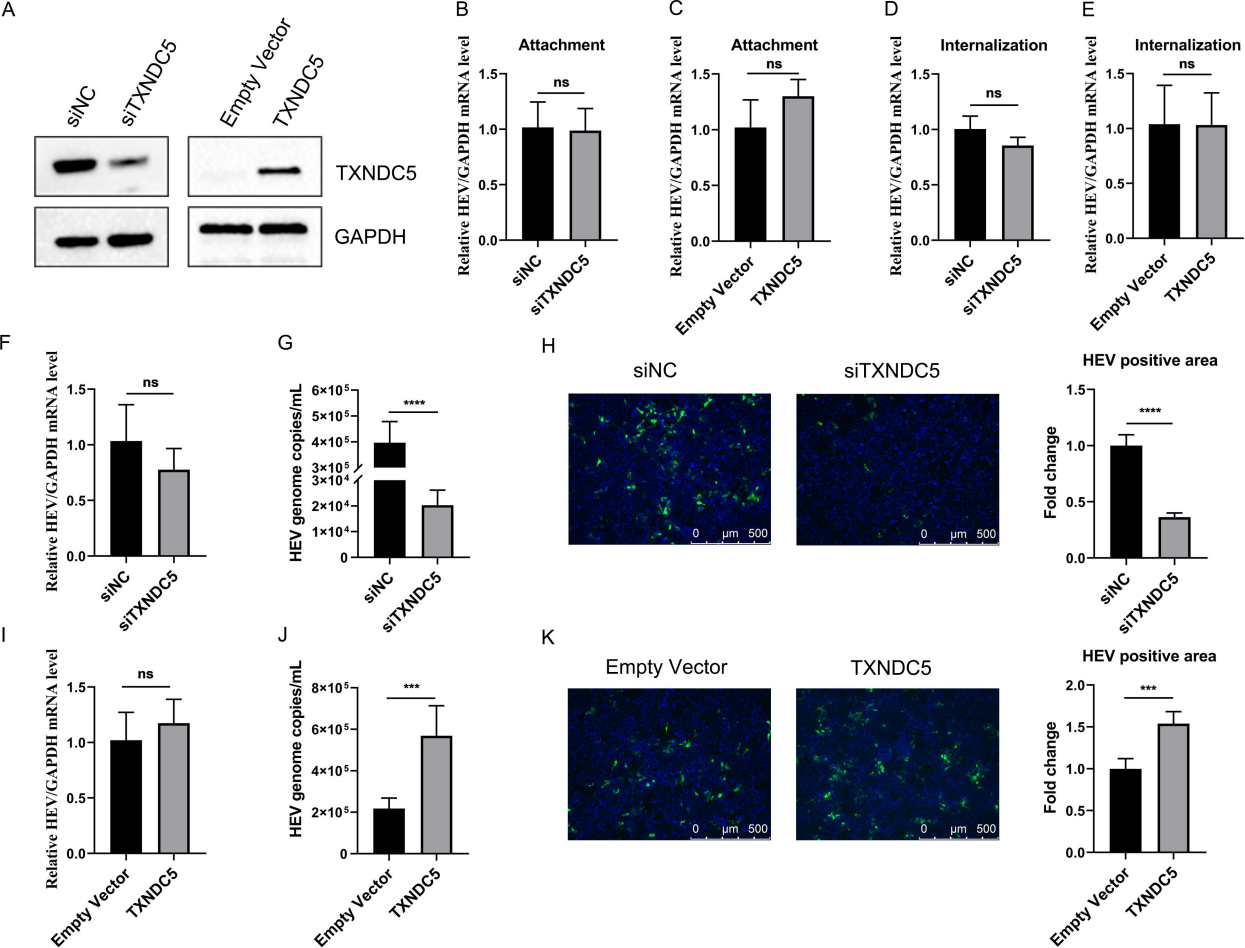
The host factor TXNDC5 is involved in HEV release from the host cells. (**A**) Western blotting analysis of TXNDC5 expression in HepG2/C3A cells transfected with siRNAs or overexpressed plasmids. (**B and C**) Knockdown and overexpression of TXNDC5 did not affect HEV attachment. After TXNDC5 expression was knocked down (**B**) or overexpressed (**C**), the HepG2/C3A cells were inoculated with HEV for 2 h at 4°C. After washing with cold PBS to remove the unbound viruses, the RNA copies of HEV attaching the cells were analyzed by RT-qPCR. (**D and E**) Knockdown and overexpression of TXNDC5 did not affect HEV internalization. After the attachment assay, the knocked down (**D**) or overexpressed (**E**) cells were further incubated with HEV for 2 h at 37°C, and then, the HEV RNA in the cells was detected by RT-qPCR. (F–H) Knockdown of TXNDC5 did not affect HEV genome replication and decreased HEV particle release. The HepG2/C3A-p6 cells were transfected with siTXNDC5 or siNC for 48 h at 37°C. The intracellular HEV RNA was relatively quantified by qPCR with normalization to GAPDH (**F**), and HEV RNA in supernatants was estimated by absolute quantitative RT-PCR using the standard plasmid containing part of the ORF1 gene (**G**). (**H**) The supernatants were further inoculated into the HepG2/C3A cells for 7 d, and then, the HEV-positive cells were stained with 1E6 mAb. HEV-positive areas were quantified by ImageJ software. (I–K) Overexpression of TXNDC5 increased HEV particle release. The HepG2/C3A-p6 cell model was transfected with TXNDC5 or empty vector for 48 h at 37°C, and the intracellular compartments (**I**) and supernatants (**J**) were collected and used to detect HEV RNA by RT-qPCR. (**K**) The supernatants were further used to infect HepG2/C3A cells for 7 d and detected by indirect immunofluorescence assay (IFA) using 1E6 mAb. HEV-positive areas were quantified by ImageJ software. Scale bars indicated 500 µm. The data are presented as the means ± SDs from five independent experiments. Statistical analysis was carried out using Student’s *t*-test. ^***^P < 0.001; ^****^P < 0.0001.

To determine the functions of TXNDC5 in different stages of HEV infection, TXNDC5 was knocked down or overexpressed in HepG2/C3A cells. Firstly, Western blotting showed that TXNDC5 had been knocked down by siRNA and overexpressed by recombinant plasmid transfection (Fig. 3A). Next, the knocked down and overexpressed HepG2/C3A cells were inoculated with Kernow-C1/p6 at 4°C for 2 h. After adsorption, cold phosphate-buffered saline (PBS) washed the cells, and the copies of the HEV genome were analyzed by RT-qPCR. The results showed that there was no significant difference in HEV RNA abundance between siNC- and siTXNDC5-transfected cells (Fig. 3B). Furthermore, the overexpression of TXNDC5 cannot influence HEV attachment (Fig. 3C). Subsequently, the effect of TXNDC5 on HEV internalization was determined. The siRNA- or plasmids-transfected HepG2/C3A cells were inoculated with HEV at 4°C for 2 h, and then, the cells were moved to 37°C for another 2 h. After washing with PBS, the HEV RNA from the cells was detected by RT-qPCR. The results showed that knockdown or overexpression of TXNDC5 in HepG2/C3A cells cannot affect amounts of HEV RNA (Fig. 3D and E). Collectively, TXNDC5 expression did not affect HEV attachment and internalization.

Additionally, whether TXNDC5 is involved in HEV genome replication and release was further determined. The HepG2/C3A cells’ insistent propagation of Kernow-C1/p6 (g3-HEV) was transfected with siRNA for 48 h. The HEV RNA in intracellular and extracellular compartments from siNC- or siTXNDC5-transfected cells was analyzed by RT-qPCR. HEV genome from intracellular compartments of both cells showed similar amounts and no significant difference (Fig. 3F). However, the HEV RNA in the supernatants of siTXNDC5-transfected cells was significantly lower than that in siNC-transfected cells (Fig. 3G). Then, the infectivity of HEV in extracellular compartments was further assessed by inoculating the naïve HepG2/C3A cells. Supernatants collected from siTXNDC5-transfected cells led to a reduction of HEV ORF2-positive cells (Fig. 3H). Furthermore, the qPCR results also indicated that overexpression of TXNDC5 did not affect HEV genome replication (Fig. 3I) but increased HEV release (Fig. 3J). In addition, the IFA results revealed that the supernatants from TXNDC5-overexpressed cells increased the HEV-positive cells (Fig. 3K). Hence, TXNDC5 did not influence viral genome replication but affected HEV release.

### Mutant HEV ORF3 protein not interacting with TXNDC5 reduced viral particle release

**Fig 4:**
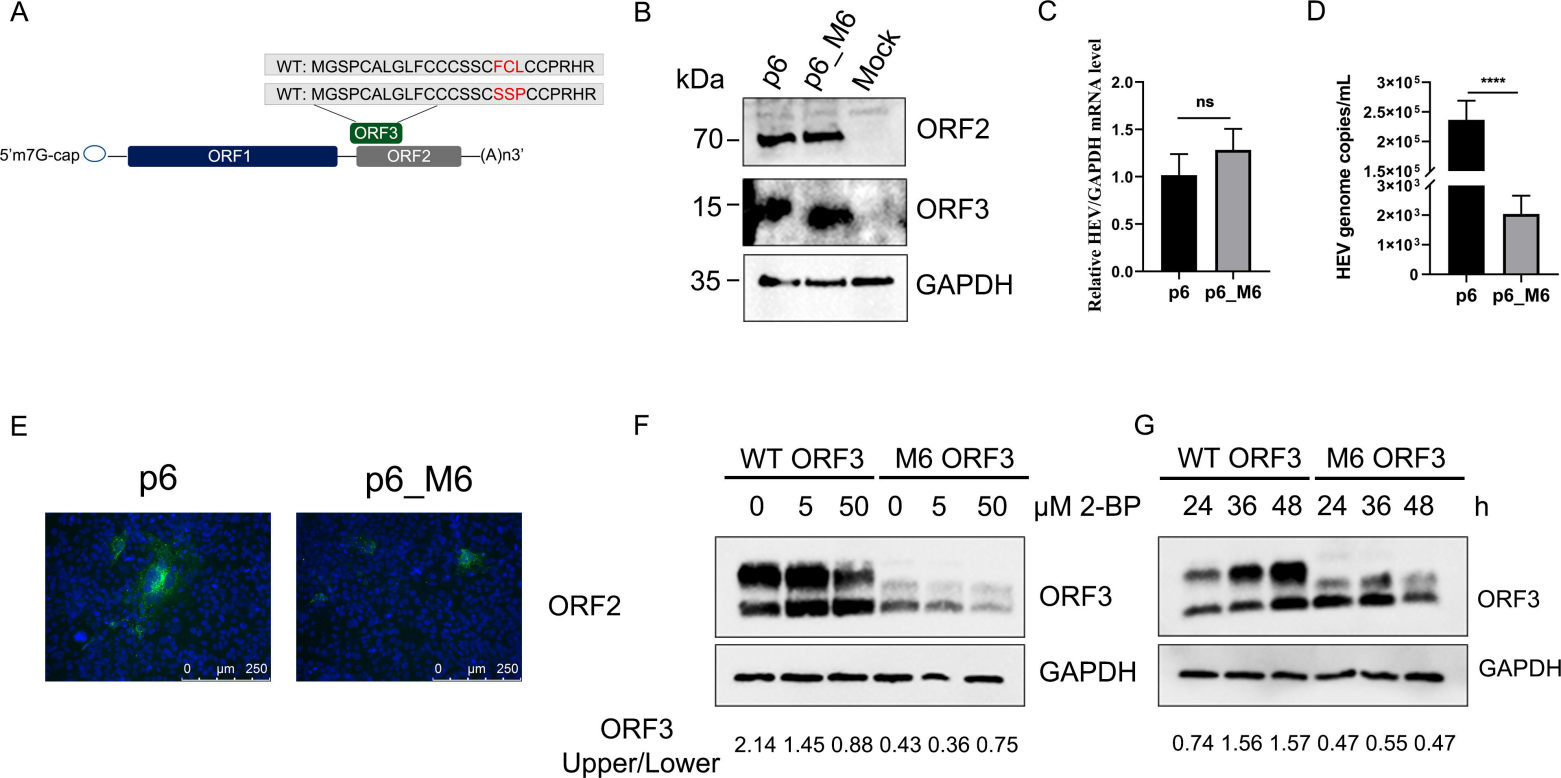
The ^17^FCL^19^ motifs of HEV ORF3 protein are essential for viral particle release. (**A**) Schematic diagram of HEV p6 and p6_M6 infectious clones. (**B**) The S10-3 cells were transfected with *in vitro* transcribed HEV p6 or p6_M6 RNA for 7 d. Then, the HEV ORF2 and ORF3 proteins were detected by Western blotting with anti-ORF2 or ORF3 protein antibodies, respectively. (**C**) There is no difference in HEV RNA copies in S10-3 cells transfected with *in vitro* transcribed HEV p6 or p6_M6 RNA. (**D**) Compared with HEV p6, the HEV RNA copies in supernatants were decreased in cells transfected with *in vitro* transcribed HEV p6_M6. (**E**) Infectivity of extracellular viral particles. The supernatants from the S10-3 cells transfected with p6 or p6_M6 RNA were inoculated with the S10-3 cells for another 7 d. Then, the cells were detected by IFA with an anti-ORF2 protein antibody. Scale bars indicated 250 µm. (**F**) Western blotting analysis showed that the palmitoylated bands (higher MW) of wild-type (WT) ORF3 protein were gradually reduced, and both palmitoylated and non-palmitoylated (lower MW) bands of M6 ORF3 were decreased. (**G**) Western blotting results suggested that the palmitoylated band of WT ORF3 protein was accumulated with time dependence, while M6 did not. The data are presented as the means ± SDs of five independent experiments. The statistical analysis was carried out using Student’s *t*-test. ^****^P < 0.0001.

To address whether TXNDC5 is involved in HEV release by interacting with ORF3 protein, the mutant HEV p6_M6 infectious clone was generated. Based on the above results about the M6 ORF3 protein not interacting with TXNDC5 (Fig. 2F), the mutant HEV p6_M6 infectious clone was designed. Due to ORF2 and ORF3 sequences overlapping in the HEV genome, the mutant HEV p6_M6 infection clone was designed to allow minimal amino acid changes in the ORF3 protein and not to cause amino acid changes in the ORF2 protein. Therefore, amino acids at positions 17–19 of ORF3 protein were substituted by ^17^SSP^19^ (Fig. 4A). For the mutation, the nucleotide sequences of ORF2 are TTCCTCCCC in the p6_M6 clone, and the amino acid sequences of ^13^FLP^15^ in ORF2 protein were not changed. Then, S10-3 cells were transfected with *in vitro* transcribed HEV p6 or p6_M6 RNA. After 7 d, the expression of ORF2 (capsid protein) or ORF3 protein was analyzed by Western blotting. HEV p6 RNA- and p6_M6 RNA-transfected cells had comparable amounts of ORF2 and ORF3 proteins (Fig. 4B). Meanwhile, the qPCR results revealed similar HEV RNA levels in the intracellular compartment of both transfected cells (Fig. 4C). However, compared to the HEV p6-transfected cells, the HEV RNA levels from the extracellular compartment in HEV p6_M6-transfected cells were significantly decreased (Fig. 4D). Subsequently, the S10-3 cells were then inoculated with the extracellular compartments to confirm the infectivity. As shown in Fig. 4E, compared to the HEV p6-transfected cells, the positive cells inoculated with the supernatants from HEV p6_M6 RNA-transfected cells were reduced.

### Lower stability of mutant HEV ORF3 protein in the cells

Previously, it was documented that the palmitoylation occurred in the N-terminal region of ORF3 protein and was involved in HEV release (13). Then, the palmitoylation of M6 ORF3 protein was analyzed. The cells that were expressed with WT and M6 ORF3 proteins were treated with palmitoylation inhibitor 2-bromopalmitate (13) and then analyzed by Western blotting. The results showed that the palmitoylated bands (higher MW) of WT ORF3 protein were gradually degraded, and the non-palmitoylated bands (lower MW) were accumulated (Fig. 4F). But both palmitoylated and non-palmitoylated bands of M6 ORF3 protein were reduced (Fig. 4F). These results suggested that ^17^FCL^19^ motifs might be important for the stability of ORF3 protein. Furthermore, the WT and M6 ORF3 proteins were both expressed in HEK293T cells and collected at different times. Western blotting results showed that the levels of WT ORF3 protein were accumulated in a time-dependent manner, while M6 ORF3 protein did not (Fig. 4G). Collectively, although the M6 mutant is still palmitoylation-modified, the level of palmitoylated M6 mutant is clearly reduced compared to the one of palmitoylated WT ORF3 protein. And the stability is lower than that of the WT ORF3 protein.

### HEV ORF3 protein hijacks TXNDC5 to maintain its stability in the cells

**Fig 5:**
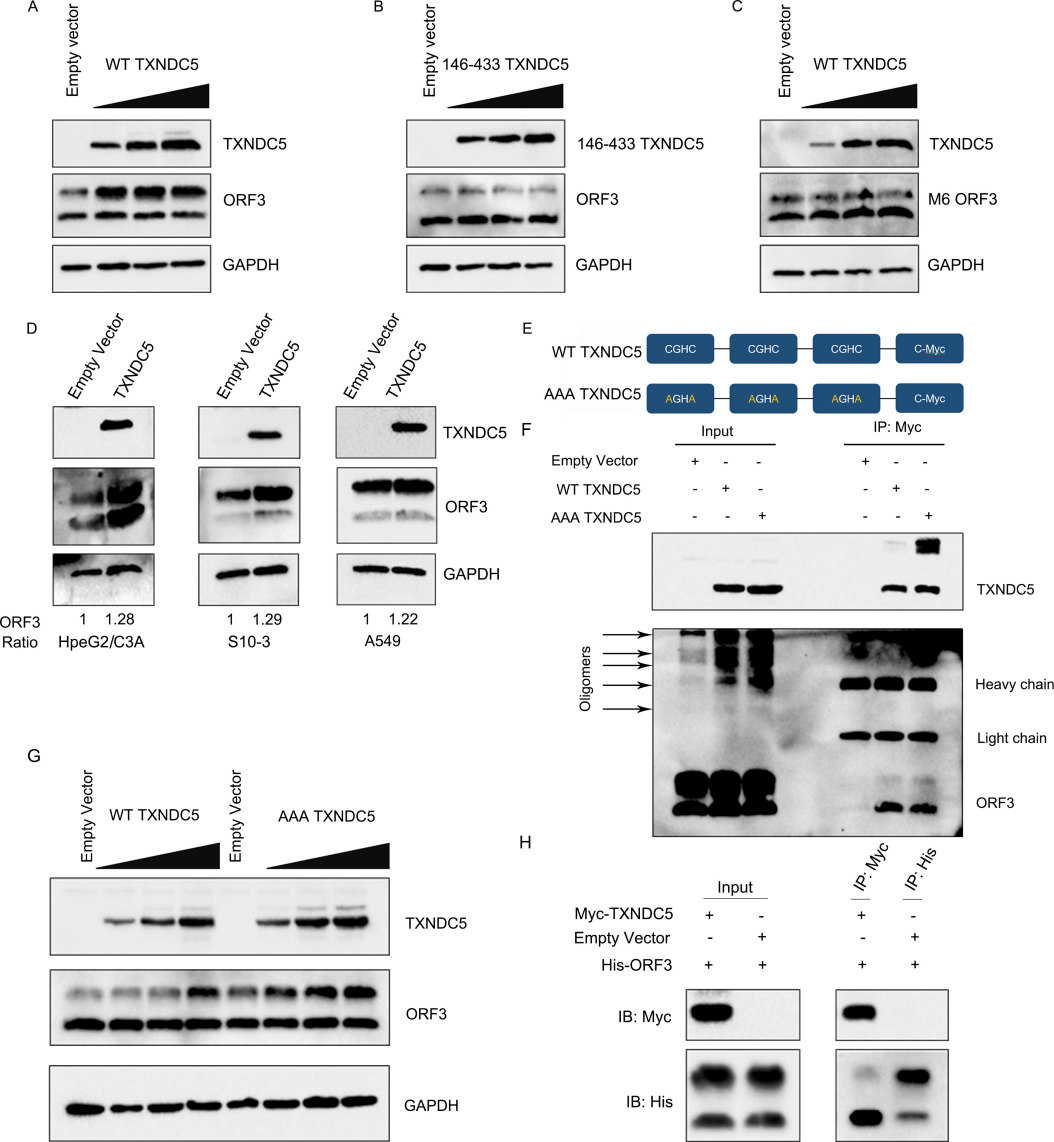
TXNDC5 maintains the stability of HEV ORF3 protein by interacting with non-palmitoylated ORF3 protein. (**A**) TXNDC5 increased the levels of ORF3 protein. HEK293T cells were co-transfected with 1 µg of ORF3 expression plasmids and increasing amounts of TXNDC5 expression vector (0.5, 1, and 2 µg) or empty vector. After 20 h post-transfection, the amounts of ORF3 protein were analyzed by Western blotting. (**B**) TXNDC5 (146-433) mutant cannot increase ORF3 protein levels. HEK293T cells were co-transfected with 1 µg of ORF3 expression plasmids and increasing amounts of TXNDC5 (146-433) mutant expression vector (0.5, 1, and 2 µg) or empty vector. The cells were collected at 20 h post-transfection and analyzed by Western blotting. (**C**) TXNDC5 cannot increase the levels of M6 ORF3 protein. HEK293T cells were co-transfected with 1 µg of M6 ORF3 expression plasmids and increasing amounts of TXNDC5 expression vector (0.5, 1, and 2 µg) or empty vector. After 20 h post-transfection, the M6 ORF3 protein was assessed by Western blotting. (**D**) TXNDC5 increased the levels of ORF3 protein in hepatoma and human lung carcinoma cell lines. TXNDC5 expression plasmids were co-transfected with ORF3 expression plasmids in HepG2/C3A cells, S10-3 cells, and A549 cells. After 48 h post-transfection, the cells were analyzed by Western blotting. (**E**) Schematic diagram of TXNDC5 or its cysteine-specific mutants. (**F**) Identification of the interaction between WT-TXNDC5 and AAA-TXNDC5 with ORF3 protein. The ORF3 protein can be pulled by WT-TXNDC5 or AAA-TXNDC5. (**G**) Amount analysis of ORF3 protein in cells co-transfected with ORF3 and WT-TXNDC5 or AAA-TXNDC5. Both WT TXNDC5 and AAA TXNDC5 can maintain the stability of ORF3 protein. (**H**) TXNDC5 interacted with the non-palmitoylated ORF3 protein (lower MW band). HEK293T cells were co-transfected with His-ORF3 expression plasmids and Myc-TXNDC5 expression plasmids or empty vector, and then, the cell lysates were immunoprecipitated with anti-Myc mAb or anti-His mAb and analyzed by Western blotting with the indicated antibodies.

To further analyze whether the TXNDC5 can stabilize ORF3 protein in the cells, the increasing amounts of TXNDC5 plasmids were co-transfected with a constant amount of ORF3 ones in HEK293T cells. The results of Western blotting showed that the levels of ORF3 protein increased during the increasing amount of TXNDC5 in the cells (Fig. 5A). However, the truncated TXNDC5 (146-433 TXNDC5), which cannot bind to ORF3 protein, lost the ability to increase ORF3 protein amounts (Fig. 5B). Additionally, the levels of M6 ORF3 protein cannot be increased by the TXNDC5 (Fig. 5C). Next, we analyzed whether the TXNDC5 activity is cell type-specific. It was previously reported that HEV can propagate in hepatoma and human lung carcinoma cells. Here, HepG2/C3A, S10-3, and A549 cell lines could support HEV infection (35) and were used to study the effect of TXNDC5 on the stability of ORF3 protein. Due to the transfection and expression efficiency, cells were harvested in these cells after 48 h post-transfection. The results showed that the amounts of ORF3 protein were also increased in all overexpressed TXNDC5 cells (Fig. 5D), suggesting that HEV ORF3 protein may hijack TXNDC5 to maintain its stability in HepG2/C3A, S10-3, and A549 cell lines.

Like most members of the PDI family, TXNDC5 can catalyze the formation and rearrangement of disulfide bonds through its disulfide isomerase activity that functions by the CXXC motif of the Trx-like domain. Therefore, we further determined the effect of TXNDC5 disulfide isomerase activity on ORF3 protein stability. Firstly, the AAA-TXNDC5 mutant with two cysteine residues in each Trx-like domain mutated to alanine (CGHC to AGHA) was synthesized (Fig. 5E). Next, the interaction between AAA-TXNDC5 and ORF3 protein was analyzed by the Co-IP assay. The results showed that the mutation of the TXNDC5 Trx-like domain can still interact with ORF3 protein (Fig. 5F). Then, the plasmids containing the ORF3 gene were co-transfected with increasing amounts of AAA-TXNDC5 mutant. The results showed that the levels of ORF3 protein still gradually increased (Fig. 5G), suggesting that TXNDC5 stabilized ORF3 protein independently of its disulfide isomerase activity.

Based on the above interaction results, they all showed that the non-palmitoylated (lower MW) band of ORF3 protein was pulled by the TXNDC5 (Fig. 2B), indicating that non-palmitoylated ORF3 protein may hijack TXNDC5 to maintain its stability. Then, to confirm the results further, TXNDC5 was used to pull down ORF3 protein by the Co-IP assay with anti-Myc mAb. Meanwhile, the ORF3 protein with His tag was directly pulled down with anti-His mAb as controls. Western blotting results showed that the TXNDC5 is associated with the non-palmitoylated band of ORF3 protein, and two bands were both pulled down by anti-His mAb (Fig. 5H). These results suggested that the HEV ORF3 protein hijacked TXNDC5 for its stability through its non-palmitoylated forms interacting with TXNDC5.

### Knockdown of TXNDC5 facilitated degradation of HEV ORF3 protein

**Fig 6:**
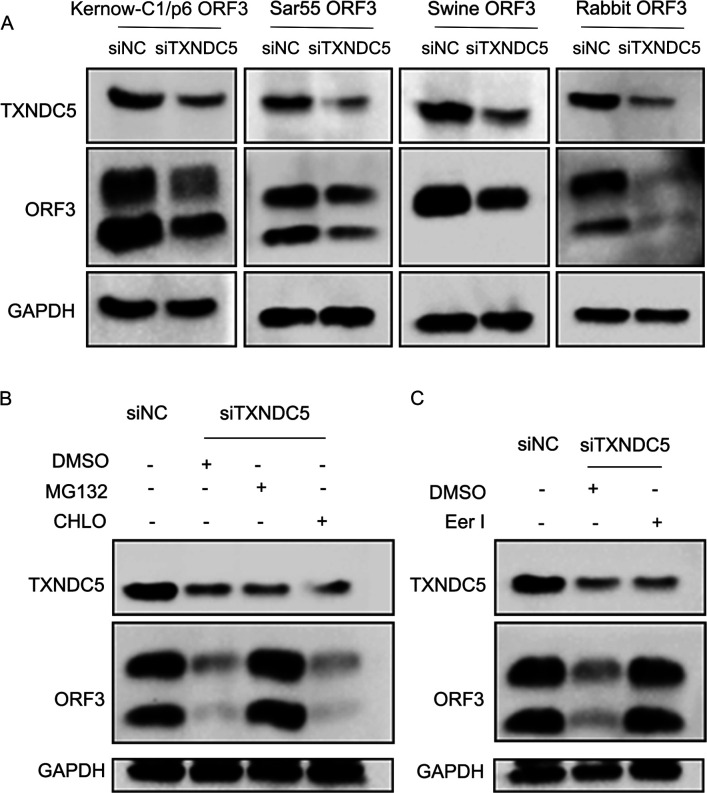
Knockdown of TXNDC5 mediated ORF3 degradation through ER-associated degradation (ERAD)-proteasome systems. (**A**) Western blotting analysis of four HEV ORF3 proteins in the A549 cells transfected with siTXNDC5. The four HEV ORF3 protein amounts were reduced in siTXNDC5-transfected cells. (**B**) The inhibitor of MG132, but not CHLO, can rescue ORF3 protein amounts in siTXNDC5-transfected cells. (**C**) The inhibitor of eeyarestatin I (Eer I) can restore ORF3 protein amounts in siTXNDC5-transfected cells.

To further confirm that HEV ORF3 protein hijacks TXNDC5 to maintain its stability, the ORF3 protein was expressed in the knockdown-TXNDC5 cells. As the knockdown efficiency of TXNDC5 was low in HEK293T cells and plasmids and transfection efficiency was extremely low in siRNA-transfected hepatoma cells, we selected A549 cells for the next study. The results showed that all the amounts of four HEV ORF3 proteins were decreased in siTXNDC5-transfected cells (Fig. 6A), suggesting that the knockdown of TXNDC5 facilitated HEV ORF3 protein degradation.

To determine which protein degradation pathway was involved in ORF3 protein, cells were treated with proteasome inhibitor MG132 and lysosome inhibitor chloroquine. The results showed that the proteasome inhibitor MG132 but not the lysosomal inhibitor chloroquine restored TXNDC5 knockdown-mediated downregulation of ORF3 expression (Fig. 6B). Subsequently, as TXNDC5 is an ER resident protein, we addressed whether ERAD was involved in ORF3 protein degradation. The cells were treated with ERAD inhibitor Eer I, which inhibits the functions of valosin-containing protein and translocation mediated by Sec61 (36). The Western blotting results showed that Eer I reversed ORF3 protein degradation in knockdown-TXNDC5 cells (Fig. 6C). These findings indicated that the knockdown of TXNDC5 may lead to ORF3 protein degradation by the ERAD-proteasome system, and TXNDC5, as a chaperone, facilitated the stability of ORF3 protein in ER.

### The PDI family is involved in HEV ORF3 expression

**Fig 7:**
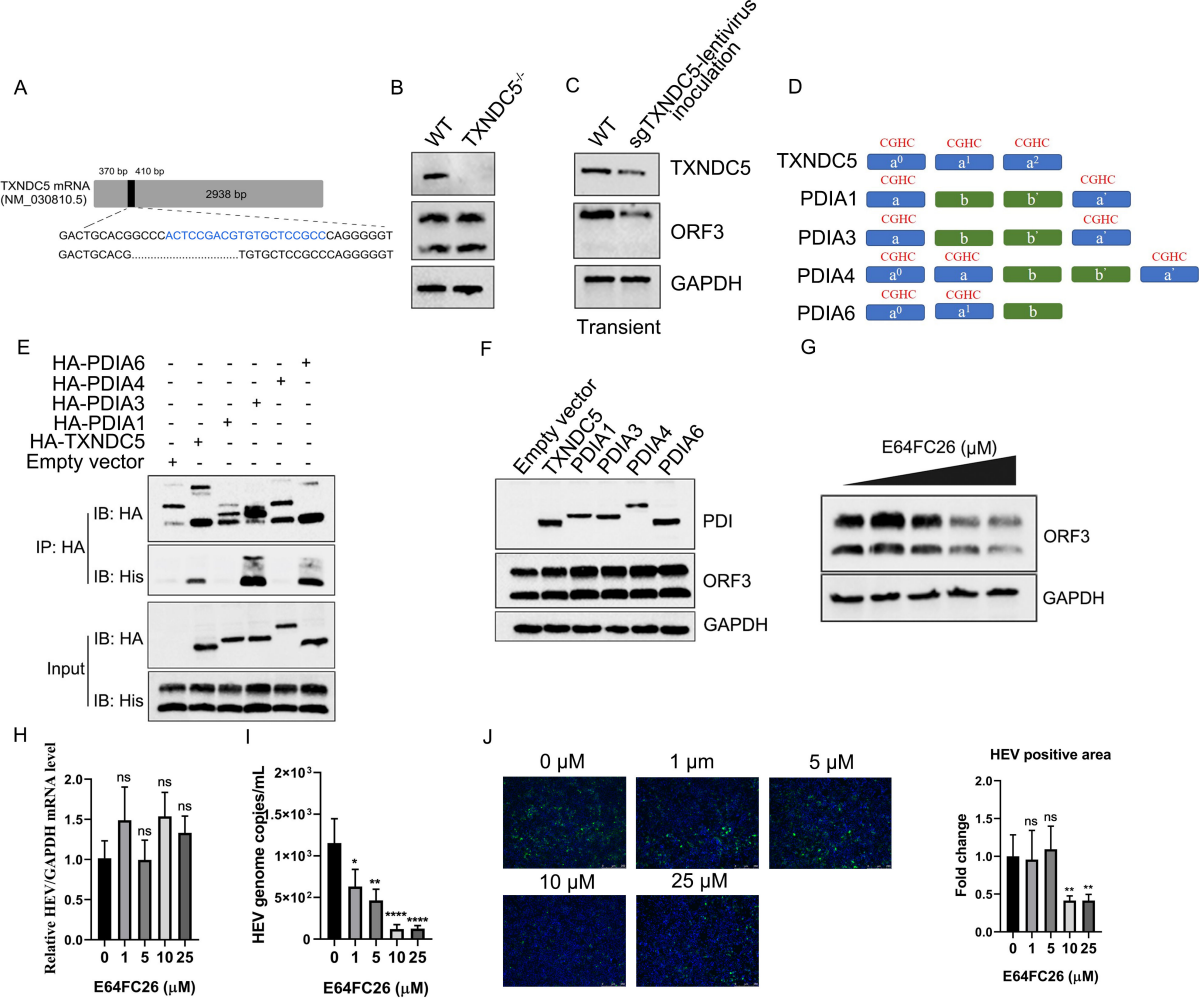
PDIA1, PDIA3, PDIA4, and PDIA6 are involved in the stability of HEV ORF3 protein in the cells. (**A**) Construction of the A549 cell lines knocked out TXNDC5 using the CRISPR/Cas9 system. Sequence analysis of A549-WT and A549-TXNDC5^−/−^ cell lines. (**B**) Western blotting analysis of ORF3 proteins expressed in the A549-WT and A549-TXNDC5^−/−^ cell lines. There was no difference in ORF3 protein levels of the two cell lines. (**C**) The A549 cells were inoculated with sgTXNDC5 lentivirus for 24 h and then transfected to express ORF3 proteins. The levels of ORF3 protein were reduced in sgTXNDC5-inoculated cells. (**D**) Schematic diagrams of five PDI family members. The motifs of Trx-like domains are indicated. (**E**) Identification of the interaction between PDIs and HEV ORF3 protein. HEV ORF3 protein can be pulled out by the TXNDC5, PDIA3, and PDIA6. (**F**) Amount analysis of HEV ORF3 protein in the ORF3 and TXNDC5, PDIA1, PDIA3, PDIA4, and PDIA6 co-expressed HEK293T cells. The levels of HEV ORF3 protein increased in the overexpressed cells of TXNDC5, PDIA1, PDIA3, PDIA4, and PDIA6. (**G**) The inhibitor E64FC26 of the PDI family can significantly decrease the amounts of HEV ORF3 protein in the HEK293T cells. (**H**) There is no difference in HEV RNA copies in the intracellular compartments of HepG2/C3A-p6 cells treated with the different amounts of inhibitor E64FC26. The HEV RNA in the intracellular compartments was quantified by qPCR with normalization to GAPDH. (**I**) There was a decrease of HEV RNA copies in the supernatants of HepG2/C3A-p6 cells treated with the different amounts of inhibitor E64FC26. The HEV RNA was determined with qPCR by comparing it with the standard plasmid containing part of the ORF1 gene. (**J**) Infectivity analysis of extracellular viral particles. The supernatant from E64FC26-treated HepG2/C3A-p6 cells was inoculated with HepG2/C3A cells for 7 d and detected by IFA with anti-ORF2 antibody. The HEV-positive areas were quantified by ImageJ. Scale bars indicated 250 µm. The data are presented as the means ± SDs of five independent experiments. The statistical analysis was carried out using Student’s *t*-test. ^*^P < 0.05; ^**^P < 0.01; ^****^P < 0.0001.

To analyze whether the ORF3 protein can be degraded in the knockout-TXNDC5 cells, the stable cell lines of A549-TXNDC5^−/−^ were designed and generated using the CRISPR/Cas9 system (Fig. 7A). The ORF3 protein was expressed in A549-WT and A549-TXNDC5^−/−^ cell lines, respectively. However, not as expected, the Western blotting results showed comparable amounts of ORF3 protein in the two cells (Fig. 7B), which were different in the knockdown-TXNDC5 cells. We speculated that while obtaining the A549-TXNDC5^−/−^ stable cell lines, the cells have been passaged for 10 generations, and other PDIs from the PDI family might replace TXNDC5 functions to maintain cell activity. However, the siRNA was transiently transfected to obtain the knockdown-TXNDC5 cells, and the other members of the PDI family cannot immediately compensate for TXNDC5 functions in the cells. To verify this speculation, the cells were inoculated with sgTXNDC5 lentivirus for 24 h and then transfected for expressing ORF3 protein. The results showed that the TXNDC5 was knocked down by about 40%, while the ORF3 protein was decreased by about 50% (Fig. 7C). Additionally, we analyzed the data from LC-MS/MS and found that PDIA1 (PDI), PDIA3 (ERp57), PDIA4 (ERp70), and PDIA6 (ERp5) were pulled down by ORF3-Fc as well as Fc protein (Fig. 7D). Because these PDIs were also pulled down by only Fc tag, they were not selected from the data of LC-MS/MS. Next, the Co-IP assay with ORF3 protein only fused with His tag was used to confirm the interaction between ORF3 and the above PDIs. The Co-IP results indicated that the PDIA3 and PDIA6 showed a robust interaction with ORF3 protein (Fig. 7E). Next, the five PDI members were co-expressed with ORF3 protein, respectively. Compared with empty vector, PDIA1, PDIA3, PDIA4, and PDIA6 can also increase the amounts of ORF3 protein (Fig. 7F). Meanwhile, because there are no inhibitors for specifically targeting TXNDC5, the inhibitor E64FC26 (37), which inhibits the activity of PDIA1, PDIA3, PDIA4, PDIA6, and TXNDC5, was used to treat cells. The results showed that inhibiting the activity of the five PDIs could lead to the degradation of the ORF3 protein (Fig. 7G). Moreover, the effect of E64FC26 on Kernow-C1/p6 release was evaluated. The HepG2/C3A cells infected with Kernow-C1/p6 were treated with E64FC26 for 8 h. Then, the HEV RNA in intracellular compartments and extracellular compartments was detected by qPCR. The results showed that the HEV RNA in the intracellular compartments of E64FC26-treated cells revealed no significant difference (Fig. 7H). However, the HEV RNA in the supernatants of E64FC26-treated cells decreased dose-dependently (Fig. 7I). Additionally, HEV infectivity in extracellular compartments was assessed by inoculating the HepG2/C3A cells. Supernatants collected from E64FC26-treated cells led to a reduction of HEV ORF2-positive cells (Fig. 7J).

## DISCUSSION

**Fig 8:**
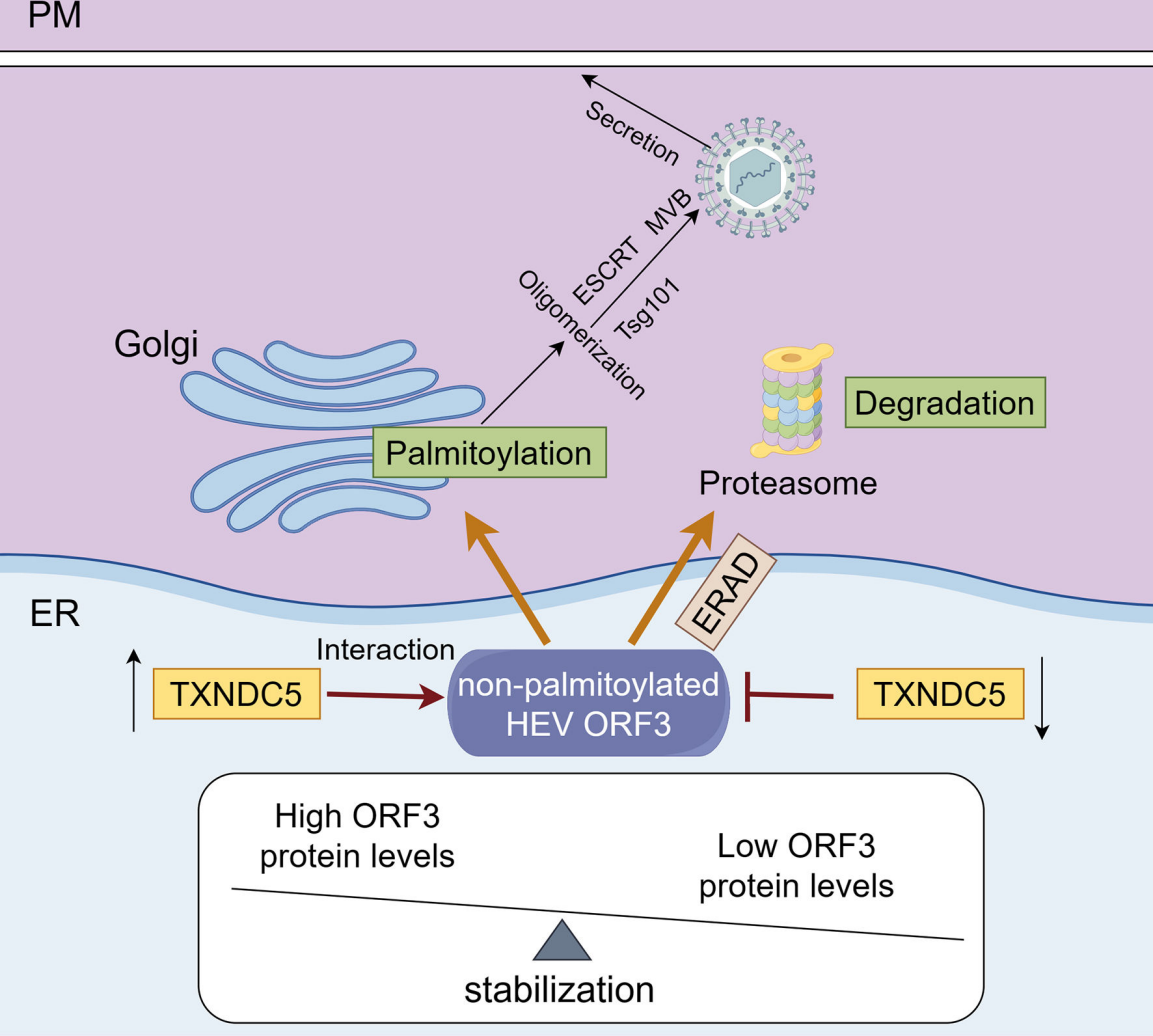
Schematic model of TXNDC5 maintaining the stability of ORF3 protein in the host cells to facilitate HEV release. TXNDC5 maintains the stability of ORF3 protein in the ER by interacting with the non-palmitoylated ORF3 protein, and following is that the ORF3 proteins were transported into the Golgi apparatus (GA) and underwent a series of modifications to promote the secretion of the virus. However, the ORF3 protein is degraded in the knockdown-TXNDC5 cells through the ERAD-proteasome pathway. This figure was drawn by Figdraw.

HEV is a zoonotic virus and is mainly transmitted through fecal–oral routes. It is a serious global public health problem, resulting in an estimated 20 million people infected with HEV (38). Due to the lack of highly efficient cell lines for HEV infection *in vitro*, the life cycle of HEV still needs to be clarified. This study identified a novel host protein, TXNDC5, interacting with ORF3 protein, essential for HEV release from the cells. We revealed that HEV ORF3 protein hijacks the TXNDC5 to maintain its stability in the endoplasmic reticulum (Fig. 8). Then, the following modification of ORF3 protein can continue to help viral release. However, the ORF3 protein cannot be degraded in the knockout-TXNDC5 stable cell lines, suggesting that it may hijack other proteins for its stabilization. Subsequently, we found that PDIA1, PDIA3, PDIA4, and PDIA6 can also increase the levels of ORF3 protein in the cells, while PDIA3 and PDIA6 can interact with ORF3 protein. As we know, it is the first time to document that TXNDC5 maintains the stability of viral proteins and facilitates viral particle release.

Increasing evidence indicated that ORF3 protein is critical for HEV release (39). The protein can form multimeric complexes with ion channel activity and associate with HEV release (16). Additionally, the absence of ORF3 did not affect the HEV attachment, internalization, and genome replication but impaired HEV release (17, 40). In the present study, TXNDC5 was determined to interact with ORF3 protein. Then, based on the function of ORF3 protein in the HEV life cycle, the function of TXNDC5 was focused on HEV release. Because the efficiency of HEV genome replication and release *in vitro* is too low, HepG2/C3A-p6 cell lines stably propagating HEV were used to verify the HEV release (a gift from Suzanne U. Emerson) (41). In this cell model, we determined that the overexpression or knockdown of TXNDC5 positively regulated HEV release but did not influence HEV genome replication (Fig. 3). In the future, more experiments will be needed to clarify the reasons for the HepG2/C3A-p6 cell lines stably propagating HEV.

TXNDC5 could bind to peptides containing aromatic and basic residues by its catalytic domains for catalyzing substrate (42), which was different from the other members of the PDI family (32). Here, we determined that TXNDC5 cannot interact with ORF3 protein during ^17^FCL^19^ mutation in ORF3 protein (Fig. 2F). The motif ^17^F of ORF3 protein was just the right aromatic amino acid, which confirmed the description of reference (42). Interestingly, our results also showed that the interactions between TXNDC5 and ORF3 M2 and M3 mutants were even more vital than the ones of the WT ORF3 protein. We thought that the ORF3 M2 and M3 mutants may change the structure of the ORF3 protein, which enhances the interaction with TXNDC5. More experiments will be performed to clarify the interesting results. In the present study, we mainly focused on the ORF3 M6 mutants, which did not interact with TXNDC5.

Gouttenoire et al. previously showed that the N- and C-terminal regions of ORF3 protein are membrane-associated and localized in the cytoplasm (13). They also indicated that palmitoylation of HEV ORF3 protein determines its membrane association and subcellular localization. However, in the study, the Co-IP and confocal experiment results showed that the N-terminal region of ORF3 protein was co-localized with TXNDC5 (Fig. 2D and K), indicating that ORF3 protein was present in the ER lumen and may adopt different membrane topology and subcellular localization in the replication cycle. Additionally, we also found that the TXNDC5 was identified to interact with non-palmitoylated ORF3 protein (Fig. 5H). Then, based on the results described by Gouttenoire et al. and us, we speculated that the non-palmitoylated ORF3 protein may be located in the ER lumen and the palmitoylated ORF3 protein is membrane-associated and localized to intracellular membranes. In the future, more experiments will be needed to confirm our speculations.

As a member of the PDI family, TXNDC5 has disulfide isomerase activity that can catalyze disulfide bond rearrangement but is also a chaperone protein that does not rely on isomerase and functional domain activity (33). TXNDC5 plays an important role in organ fibrogenesis by folding/stabilizing extracellular matrix (ECM) and TGFβR1 proteins and triggering TGFβ-induced fibrogenic response (43–45). In the present study, we found that HEV ORF3 protein, as a foreign protein of host cells, can also hijack TXNDC5 to maintain its stability. Overexpression of TXNDC5 could increase the levels of ORF3 protein (Fig. 5A). Of note, the AAA-TXNDC5 mutant, lacking the disulfide isomerase activity, can also interact with ORF3 protein and increase the levels of ORF3 protein (Fig. 5F and G). Therefore, TXNDC5 acts as a chaperone protein independent of its disulfide isomerase activity to maintain the stability of the ORF3 protein. As we know, the palmitoylated ORF3 protein is mainly modified in the GA (46).

Interestingly, our results showed that the TXNDC5 interacted with the non-palmitoylated ORF3 protein (lower MW band) in the ER (Fig. 5H). Therefore, we thought that the ORF3 protein hijacked the TXNDC5 in the ER for its stability and then transported it to the Golgi apparatus for palmitoylated modification. Furthermore, the multimeric form is another characteristic of HEV ORF3 protein by detecting Western blotting (16). We found that the overexpression of WT-TXNDC5 and AAA-TXNDC5 can increase the oligomer amounts of ORF3 protein in the cells (Fig. 5F), which indicates that ORF3 protein hijacking the TXNDC5 can maintain its stability, following its modification and multimeric form assembly.

The ER mainly contains two systems for protein degradation: one is ERAD, which specifically degrades misfolded proteins through the ubiquitin-proteasome system, and another is reticulophagy, which degrades protein aggregates through the autophagy-lysosome system (47). Previous studies have documented that the knockdown of TXNDC5 can result in the degradation of ECM protein by the ERAD system and cause the degradation of TGFBR1 protein via the proteasome system (43, 44). Here, we first identified that knockdown of TXNDC5 can lead to the degradation of HEV ORF3 protein. Our findings also indicated that HEV ORF3 protein, as a foreign protein for the host cells, can hijack TXNDC5 to maintain its stability. If the expression of TXNDC5 was knocked down, ERAD-proteasome systems degraded the HEV ORF3 protein, and then, the amounts of the following modification and correct folding of ORF3 protein were also decreased. Previous studies showed that the knockdown of TXNDC5 can lead to ER stress by increasing CHOP protein expression (48, 49). Subsequently, ER stress activated the unfolded protein response (UPR) (50). Thus, for the degradation of ORF3 protein in the knockdown-TXNDC5 cells, we thought that another possibility is that the knockdown of TXNDC5 leads to ER stress activating UPR, and then, ERAD is involved in the misfolded ORF3 protein retro-translocation from the ER lumen into the cytosol for degradation by the proteasome. In the future, more experiments will be performed to analyze ORF3 protein folding forms in the knockdown-TXNDC5 cells.

In the present study, unexpectedly, the stable cell line in which TXNDC5 was knocked out did not affect the expressing levels of ORF3 protein (Fig. 7B), which was not consistent with the results in the knockdown-TXNDC5 cells (Fig. 6A). We speculated that the inconsistent results were due to the stable and transient transfection cell lines. The A549-TXNDC5^−/−^ stable cell lines were obtained by passaging for 10 generations, and then, they adapted to the absence of TXNDC5. We thought that in the A549-TXNDC5^−/−^ stable cell lines, other PDIs might replace its functions to maintain cell activity. Then, when the ORF3 protein is expressed in the cell lines, it may also hijack the other members of the PDI family, replacing TXNDC5 for its stabilization in the cells. However, in the transient transfection cell lines (siRNA and sgTXNDC5 lentivirus knockdown cells), the ORF3 protein cannot immediately hijack other members of the PDI family (Fig. 7C). Subsequently, we confirmed that HEV ORF3 protein can utilize the PDIA1, PDIA3, PDIA4, and PDIA6 to keep its stability in the cells and interact with PDIA3 and PDIA6. Previous studies also reported that combining PDIA1 with TXNDC5 or PDIA6 can accelerate the formation of Prx4-dependent oxidative folding (51). Furthermore, they were also reported that each PDI (PDIA1, PDIA3, PDIA4, PDIA6, and TXNDC5) could effectively increase the folding of bovine pancreatic trypsin inhibitor (BPTI) (51, 52). For virus infection, it was also reported that the replication of influenza A and B viruses relies on the functions of PDI1, PDIA3, and PDIA4 (53). Then, based on these references and our results, the follow-up works will be focused on the precise causes of the inconsistent results.

In this study, we identified that the siRNA specific to TXNDC5 can significantly reduce HEV release from the cells (Fig. 3G and H). Currently, there is no specific inhibitor targeting TXNDC5. We found that E64FC26, an inhibitor targeting PDIA1, PDIA3, PDIA4, PDIA6, and TXNDC5, can reduce HEV release (Fig. 7I and J). These results suggested that TXNDC5 may serve as a novel target for the development of HEV drugs. In addition, the PDI family as potential drug targets have also been reported in many diseases, such as cancer invasion and migration, organ fibrosis, and virus infection (54). For example, the compound juniferdin, a PDI inhibitor, can inhibit PDI-mediated HIV-1 entry (55). The PDI inhibitors, including juniferdin, LOC14, 16F16, PACMA31, isoquercetin, epigallocatechin-3-gallate, and nitazoxanide, also documented that they can significantly reduce the replication of influenza A and B viruses (25, 53).

Our results revealed that TXNDC5 contributes to HEV release by facilitating ORF3 protein stability in the endoplasmic reticulum. These results expand the understanding of HEV infection in the host cells and guide the development of novel therapeutic targets to prevent HEV infection.

## MATERIALS AND METHODS

### Cells and viruses

Human embryo kidney 293T (HEK293T), HepG2/C3A, S10-3, and A549 cell lines were from the American Type Culture Collection (56). HEK293T, A549, and S10-3 cell lines were grown in Dulbecco’s modified Eagle’s medium (Gibco, New York, United States) supplemented with 10% fetal bovine serum (FBS; Biological Industries, Kibbutz Beit Haemek, Israel) and antibiotics (100 U/mL penicillin and 100 µg/mL streptomycin). HepG2/C3A cells were cultured in minimum essential medium (MEM; Gibco) supplemented with 10% FBS and antibiotics. HepG2/C3A-p6 (GenBank accession no. JQ679013) cell lines were generously provided by Suzanne U. Emmerson (41). All cells were cultured at 37°C with 5% CO_2_.

### Antibodies and reagents

The following antibodies were used in this study: anti-GAPDH mouse monoclonal antibody, anti-His tag mAb, anti-HA tag mAb, anti-MYPT1 mAb, anti-TXNDC5 pAb, anti-DBN1 pAb, and anti-Myc tag pAb were purchased from ProteinTech Company (Chicago, IL). Anti-HEV ORF2 protein mAb (Clone no. 1E6) was purchased from Sigma-Aldrich (St. Louis, MO). The secondary antibodies for Western blotting were horseradish peroxidase (HRP)-conjugated anti-mouse or anti-rabbit IgG (Jackson ImmunoResearch Laboratories, West Grove, PA). The secondary antibodies used for IFA were Alexa Fluor 488-conjugated goat anti-mouse IgG and Alexa Fluor 546-conjugated goat anti-rabbit IgG (Thermo Fisher Scientific, Waltham, MA).

The following reagents were used in this study: MG132, chloroquine, and 2-bromohexadecanoic acid, which were purchased from Selleck Chemicals (Houston, TX). Eeyarestatin I was purchased from APExBIO (Houston, TX). E64FC26 was purchased from MedChemExpress (Monmouth Junction, NJ). NP-40 cell lysis buffer was purchased from Beyotime Biotechnology (Shanghai, China). Protease inhibitor cocktail and phosphatase inhibitor cocktail were purchased from Roche (Basel, Switzerland).

### Plasmid construction

The recombinant plasmids containing genes encoding four ORF3 proteins from HEV-1 (Sar55, AF444002), HEV-3 (Kernow-C1/p6, JQ679013), HEV-4 (CHN-SD-sHEV, KF176351.1), and rabbit HEV-3 (CHN-SX-rHEV, KX227751) with human IgG Fc tag were constructed based on the previous descriptions (57). Briefly, the four genes encoding HEV ORF3 proteins were amplified using primers HEV-ORF3-F1/R1. Meanwhile, the genes encoding IgG Fc with His tag were also amplified by PCR via primer pairs of Fc-F2/R2. Next, overlap PCR was used to amplify the fusion fragment of ORF3, human IgG Fc, and His tag with HEV ORF3-F1 and Fc-R2 primer pairs. Then, the fusion genes were cloned into the eukaryotic expression vector pCAGEN (Addgene, Watertown, MA) by digestion with *EcoR* I and *Sph* I (TaKaRa) restriction. Additionally, the vector only containing human IgG Fc with His tag was a negative control.

To identify the domain of ORF3 protein interacting with TXNDC5, the plasmids containing the genes encoding the different truncated ORF3 proteins including aa 1–70, 5–113, 24–113, 34–113, 46–113, and 94–113 with Fc and His tags were also designed and constructed. These genes were amplified from the full-length template and cloned into the pCAGEN vector.

To express the ORF3 protein only with His tag, the target genes were amplified with the primers HEV ORF3-F1/HEV ORF3-R2 using the plasmid containing the complete ORF3 gene as the template. Then, the genes were also cloned into the pCAGEN vector by digestion with the *EcoR* I and *Sph* I restriction.

To express the TXNDC5 protein, the genes encoding complete, truncated, and mutated TXNDC5 were synthesized by GENEWIZ (Suzhou, China) according to the sequences of TXNDC5 (GenBank accession no. BC001199.1). Then, the genes were also cloned into the pCAGEN vector.

Alanine substitution mutants of ORF3 M1 to M8 were generously provided by Qiang Ding (Tsinghua University at Beijing) (16).

All the primers used in this study are presented in Table S2.

### Co-immunoprecipitation and LC-MS/MS)

HEK293T cells were grown in 10 cm^2^ dishes and transfected with recombinant plasmids for 48 h. The cells were washed thrice with PBS and lysed in NP-40 cell lysis buffer (Beyotime, Shanghai, China) containing protease inhibitor cocktail and phosphatase inhibitor cocktail (Roche, Basel, Switzerland) (58) for 30 min on ice. For the Co-IP assay using HEV ORF3 proteins with Fc tag as baits, the cell lysates were directly incubated with immunoprecipitation via Dynabeads Protein G (Thermo Fisher, Waltham, MA) overnight at 4°C and washed thrice with PBS′T. Then, the pellets were resuspended by PBS and analyzed by Western blotting. Using TXNDC5 or PDIs as baits, after the plasmids were co-transfected into HEK293T cells, the cells were collected at 48 h post-transfection. Then, the cell lysates were incubated with Dynabeads Protein G, which was pre-bound by anti-Myc mAb. After being washed with PBS′T and resuspended by PBS, the immunoprecipitation complex was analyzed by Western blotting and LC-MS/MS assay. LC-MS/MS was conducted by the Q Exactive HF Orbitrap LC-MS/MS System at Applied Protein Technology (Shanghai). With the raw data, the way we screened for positive proteins was that the host proteins can be pulled out by ORF3-Fc but not by Fc tag.

### RNA interference

To knock down the expression of proteins screened from the data of LC-MS/MS, all siRNAs were designed and synthesized from Tsingke Biotechnology (Beijing, China). The HepG2/C3A-p6 cell lines were transfected with indicated siRNAs and negative siRNA (siRNA-NC) for 48 h using the Lipofectamine RNAiMAX reagent according to the manufacturer’s instructions (Thermo Fisher, Waltham, MA). Next, the amounts of mRNA were separately analyzed by qPCR. The knockdown methods were applied for further assays. The indicated siRNAs are listed in Table S3.

### *In vitro* transcription assay and viral RNA transfection

To construct the HEV mutant plasmids Kernow-C1/p6_M6, overlap PCR was used to amplify DNA fragment containing M6 mutation from full-length HEV p6 clone using p6_M6 F1/R1 and p6_M6 F2/R2. Then, the mutation fragment was cloned into p6 WT plasmid at the *Afl* II to *Pml* I restriction sites (13).

The *in vitro* transcription assay was performed as described previously (59). Briefly, the plasmids containing HEV Kernow-C1/p6 and the mutant Kernow-C1/p6_M6 genes were linearized by *Mlu* I restriction. Viral-capped RNAs were transcribed *in vitro* from linearized plasmids using AmpliCap-Max T7 High Yield (CELLSCRIPT, Madison, WI). The reaction mixtures were mixed with linearized template DNA (1 µg), 10× T7 transcription buffer, NTP, 100 mM DTT, RNase inhibitor, and T7 enzyme solution and abbreviated IVT. Next, the IVT reaction mixture was incubated at 37°C for 30 min. DNase I was added to the IVT reaction mixture to remove the template DNA and incubated at 37°C for 15 min. Finally, the viral RNA was purified by RNA extraction using RNAiso (TaKaRa, Tokyo, Japan) following the instructions.

Then, the capped RNA was transfected into S10-3 cells using the *TransIT*-mRNA transfection reagent according to the manufacturer’s instructions (Mirus Bio LLC, Madison, WI). The opti-MEM was mixed with capped RNA (1 µg), *TransIT*-mRNA reagent (2 µL), and mRNA boost reagent (2 µL). The complex was placed at room temperature (RT) for 5 min and then incubated with S10-3 for 7 d.

### Virus infection

Kernow-C1/p6 was propagated in the HepG2/C3A cells. The supernatants were collected and centrifuged at 1,000 × *g* for 20 min, and the supernatant was collected. Next, viruses were concentrated by the Virus Concentrator following the manufacturer’s instructions (Accurate Biology, Changsha, China). After centrifugation, the pellet was resuspended with 1/10 the volume of the original virus solution in sterile MEM.

The HepG2/C3A cells (4 × 10^5^/well) were seeded into 12-well plates. The cells were then inoculated with Kernow-C1/p6 (6 × 10^6^ copies/well) for 4 h at 37°C. Next, the cells were washed thrice with PBS and refreshed with MEM containing 5% FBS (wt/vol). Finally, the cells were collected at 7 d post-inoculation and applied for confocal microscopy to analyze the co-localization of TXNDC5 with HEV ORF3 protein.

### Viral attachment and internalization assay

For the HEV attachment assay, the siRNA-transfected or plasmids-transfected cells were infected with Kernow-C1/p6 (6 × 10^6^ copies/well) and incubated at 4°C for 2 h. After washing with PBS, the relative mRNA level of HEV was analyzed by RT-qPCR.

For the HEV internalization assay, the cells were inoculated with Kernow-C1/p6 at 4°C for 2 h. And then, the cells were transferred to 37°C for 2 h. After washing with PBS containing proteinase K, the entering viral RNA was quantified by RT-qPCR.

For the HEV genome replication and release assay, the HepG2/C3A-p6 cell culture model that stably propagated HEV was transfected with siRNA or plasmids for 48 h (41). After washing with PBS, the cells were analyzed by RT-qPCR. The intracellular and extracellular compartments were separately collected and centrifuged at 12,000 × *g* for 15 min. Then, the intracellular ones were used to analyze genome replication, and the supernatants were used to detect viral RNA by RT-qPCR or incubated with HepG2/C3A cells. After viral infection, the cells were stained by IFA using anti-HEV ORF2 protein mAb (1E6) as the primary antibodies.

### RT-qPCR

Total RNA was extracted using RNAiso Plus according to the manufacturer’s protocols. The cDNA was synthesized by reverse transcription for 15 min at 37°C and 15 sec at 85°C using PrimeScript RT Master Mix (TaKaRa, Tokyo, Japan). The amounts of mRNA were quantified by SYBR Green Power Mixture (with ROX) (GenStar, Beijing, China) with an Applied Biosystem StepOnePlus Real-Time PCR System (Applied Biosystems, Foster City, CA). All the specific primers are shown in the Table S4.

### Indirect immunofluorescence assay and confocal microscopy

For IFA, the cells were fixed with 4% paraformaldehyde for 20 min and permeabilized with 0.25% Triton X-100 for 15 min. After washing more than three times with PBS, the cells were blocked with a blocking buffer (Thermo Fisher Scientific, Waltham, MA) for 2 h at RT. To detect the HEV ORF2 protein, the cells were incubated with anti-1E6 mAb for 1 h at 37°C. After washing thrice with PBS, cells were incubated with Alexa Fluor 488-conjugated goat anti-mouse IgG (Thermo Fisher Scientific, Waltham, MA) for 1 h at 37°C. Next, the cells were incubated with Fluoroshield with DAPI (Sigma-Aldrich, St. Louis, MO) for 10 min at 37°C. After washing again, the cells were visualized with Leica Application Suite X (version 1.0. Leica Microsystems) (Leica, Wetzlar, Germany).

For analyzing the co-localization of TXNDC5 with ORF3 protein in HEV-infected cells and with ORF3-Fc fusion protein in overexpression cells, the fixed cells were separately incubated with anti-TXNDC5 pAb and anti-ORF3 mAb or anti-His mAb for 1 h at 37°C and followed by Alexa Fluor 546-conjugated goat anti-rabbit IgG and Alexa Fluor 488-conjugated goat anti-mouse IgG for 1 h. Finally, cells were stained with DAPI and observed with a Leica SP8 confocal system. The co-localization of TXNDC5 and ORF3 was analyzed using ImageJ software (National Institutes of Health, United States).

### Establishment of TXNDC5-deficient cell lines

As described previously, A549-TXNDC5^−/−^ cells were generated via the CRISPR/Cas9 system (60). The single-guide RNA sequence targeting the human TXNDC5 gene (5′-GGCGGAGCACACGTCGGAGT-3′) was designed and cloned into the LentiCRISPRv2 vector. The HEK293T cells produced sgTXNDC5 lentivirus and empty vector lentivirus by transfection with psPAX2, PMD2.G, and TXNDC5KO vector or empty vector. The cell supernatants containing sgTXNDC5 lentivirus or empty vector lentivirus (negative control) were then infected with target A549 cells. After 2 d post-infection, cells were screened with 10 µg/mL puromycin. Finally, the stable cell clone was selected by using the limiting dilution method. Knockout of TXNDC5 in the A549 cells was confirmed by sequence analysis and Western blotting.

### Western blotting

Cell lysates or immunoprecipitated complexes were subjected to 12% SDS-polyacrylamide gels. Then, the gel was transferred to PVDF membranes. After blocking with 1% BSA, the membranes were sequentially incubated with the primary antibodies and HRP-labeled goat anti-rabbit or goat anti-mouse antibodies. After washing with PBS′T, the membranes were visualized with the ECL reagent and imaged using a chemiluminescence imaging system (Bio-Rad, Hercules, CA).

### Statistical analysis

Data were presented as the mean ± standard deviation. The statistical significance in the two groups was determined by Student’s *t*-test using GraphPad (GraphPad Software Inc., San Diego, CA, United States). Statistical significance is indicated as follows: ^*^P < 0.05; ^**^P < 0.01; ^***^P < 0.001; ^****^P < 0.0001, and ns means no significant difference.

## Data Availability

All study data are included in the article and/or supporting information.

## SUPPLEMENTAL MATERIAL

Table S1 jvi.01649-23-s0001.xlsx The LC-MS/MS data pulled by four HEV ORF3 proteins from three genotypes (Sar55 HEV, Kernow/C1-p6, rabbit HEV, and swine HEV).

Tables S2 to S4 jvi.01649-23-s0002.docx Primers used in this study.

ASM does not own the copyrights to Supplemental Material that may be linked to, or accessed through, an article. The authors have granted ASM a non-exclusive, world-wide license to publish the Supplemental Material files. Please contact the corresponding author directly for reuse.
