# Supplementary material for: Hepatitis E virus ORF3 protein hijacking thioredoxin domain-containing protein 5 (TXNDC5) for its stability to promote viral particle release
Source: J Virol. 2024 Mar 29;98(4):e01649-23. doi: 10.1128/jvi.01649-23 (PMC11019958; doi:10.1128/jvi.01649-23)
Supplement: Tables S2 to S4 — Primers used in this study. [file jvi.01649-23-s0002.docx]

**Table S2. Primers used for plasmids construction**

| **Primers** | **Sequence (5’ –3’)** |
| --- | --- |
| CHN-SD-sHEV-ORF3-F1 | CCCGAATTCATGGAGATGCCACCATGC |
| CHN-SD-sHEV-ORF3-R1 | GAGTTCAGGTGCTGGGCACGGTGGGCATGTACTGCCTCCACCGCCACGGCGAAGCCCCAGCTGGG |
| CHN-SD-sHEV-ORF3-Fc-F2 | GTCGACCTGCCCCAGCTGGGGCTTCGCCGTGGCGGTGGAGGCAGTACATGCCCACCGTGCCCA |
| CHN-SX-rHEV-ORF3-F1 | CCCGAATTCATGGGATCGCCATGCGCC |
| CHN-SX-rHEV-ORF3-R1 | GAGTTCAGGTGCTGGGCACGGTGGGCATGTACTGCCTCCACCGCCGCGGCGCAGCCCCAGTTG |
| CHN-SX-rHEV-ORF3-Fc-F2 | GCGTCGTCGATCTACCCCAACTGGGGCTGCGCCGCGGCGGTGGAGGCAGTACATGCCCACCGTGCCCA |
| Sar-55-ORF3-F1 | CCCGAATTCATGGGTTCGCGACCATGC |
| Sar-55-ORF3-R1 | GAGTTCAGGTGCTGGGCACGGTGGGCATGTACTGCCTCCACCGCCGCGGCGCGGCCCCAGCTG |
| Sar-55-ORF3-Fc-F2 | GTAGACCTACCACAGCTGGGGCCGCGCCGCGGCGGTGGAGGCAGTACATGCCCACCGTGCCCA |
| Kernow-C1-ORF3-F1 | CCCGAATTCATGGGATCACCATGTGCC |
| Kernow-C1-ORF3-R1 | GAGTTCAGGTGCTGGGCACGGTGGGCATGTACTGCCTCCACCGCCACGGCGCAGCCCCAGCTG |
| Kernow-C1-ORF3-Fc-F2 | CCGTCGATCTGCCCCAGCTGGGGCTGCGCCGTGGCGGTGGAGGCAGTACATGCCCACCGTGCCCA |
| Fc-F1 | CCCGAATTCGGCGGTGGAGGCAGTACATGCCCACCGTGCCCA |
| Fc-R2 | CCCGCATGCCTAGTGGTGGTGGTGGTGGTGTTTACCCGGAGACAGGGA |
| p6_M6-F1 | GGCTTAAGGGTTTCTGGAAGAAGCATTCTG |
| p6_M6-R1 | TGGGGAGGAACACGAAGAACAGCAGCAGA |
| p6_M6-F2 | TCCTCCCCATGCTGCCCGCGCCACCGG |
| p6_M6-R2 | GGCACGTGAATCAACATCAGGTACAGGG |

**Table S3. The siRNA sequences in this study**

| **Name** | **5’ –3’ (sense)** | **5’ –3’ (antisense)** |
| --- | --- | --- |
| TXNDC5 | CCAAGCGAAAGACGAACUU | AAGUUCGUCUUUCGCUUGG |
| PSB6 | CGGUUUCCACAGCAUUGAA | UUCAAUGCUGUGGAAACCG |
| LMAN1 | CACUGACAGUAAUGAUCAA | UUGAUCAUUACUGUCAGUG |
| TIM8B | CCUAGACUCUCGCACUGAA | UUCAGUGCGAGAGUCUAGG |
| RCN2 | GACUGCAGGCGAUCAUAAA | UUUAUGAUCGCCUGCAGUC |
| MYPT1 | CAGUCACAGAAGACCUUUA | UAAAGGUCUUCUGUGACUG |
| MYO1D | GGAGCUCAAUUAAAGUCUU | AAGACUUUAAUUGAGCUCC |
| DREB | CGGUUGAAGGAGCAGUCUA | UAGACUGCUCCUUCAACCG |
| ATP5L | GCUCAGACUGGUAGCUUCA | UGAAGCUACCAGUCUGAGC |
| PSA3 | GCUACAACAUUCCACUAAA | UUUAGUGGAAUGUUGUAGC |

**Table S4. Primers used for RT-qPCR**

| **Primers** | **Sequence (5’ –3’)** |
| --- | --- |
| HEV-ORF1-qF | GTTGAGCAGAACCCGAAGAG |
| HEV-ORF1-qR | CGGGCTCAGTCAAGTAAAGC |
| GAPDH-qF | ACAAGGCTGGGGCTCATTTG |
| GAPDH-qR | AGGGGCCATCCACAGTCTTC |
| TXNDC5-qF | GCACTGTGTTGGCACTCACT |
| TXNDC5-qR | CTACTTCGGCGATCTTGACC |
| PSB6-qF | ACTGACAAGCTGACACCTATTCA |
| PSB6-qR | CGGTATCGGTAACACATCTCCTT |
| LMAN1-qF | AGTTGAGGTGACATTTCGAGTG |
| LMAN1-qR | AGCTGATCCAAACACAGGGC |
| TIM8B-qF | TCACTTCATGGAGTTATGTTGGG |
| TIM8B-qR | AGACAATTTTCAGTGCGAGAGTC |
| RCN2-qF | TTCAGGTCCCGGTTTGAGTCT |
| RCN2-qR | TCAAGCCTGCCATCGTTATCT |
| MYPT1-qF | AGTTAATCGGCAAGGGGTTGA |
| MYPT1-qR | ATGACCACTATTTAGCCACTGC |
| MYO1D-qF | CCTCACCTTTTTGCTATTGCGG |
| MYO1D-qR | ACTGGCTTCCGTTTTACCAGC |
| DREB-qF | CAACTGGGTGGGCGAAGAT |
| DREB-qR | TGCTGGCGTTCACGATCAC |
| ATP5L-qF | ACTACGCCAAGGTTGAGCTG |
| ATP5L-qR | GCCCCGCTTGCCTATAATCTC |
| PSA3-qF | GCTCAATCGGCACTGGGTAT |
| PSA3-qR | ACCTGCTACTGCCATTCCAAC |
